# Real-world effectiveness, long-term safety and treatment pathway integration of radium-223 therapy in patients with metastatic castration-resistant prostate cancer

**DOI:** 10.3389/fmed.2022.1070392

**Published:** 2022-12-22

**Authors:** Joe M. O’Sullivan, Rana R. McKay, Kambiz Rahbar, Karim Fizazi, Daniel J. George, Bertrand Tombal, Anja Schmall, Per Sandström, Frank Verholen, Neal Shore

**Affiliations:** ^1^Patrick G. Johnston Centre for Cancer Research, Queen’s University Belfast and Northern Ireland Cancer Centre, Belfast, United Kingdom; ^2^Division of Hematology-Oncology, Department of Medicine, University of California, San Diego, La Jolla, CA, United States; ^3^Department of Nuclear Medicine, University of Münster Medical Center, Münster, Germany; ^4^Institut Gustave Roussy, University of Paris-Saclay, Villejuif, France; ^5^Duke University School of Medicine, Durham, NC, United States; ^6^Division of Urology, Institut de Recherche Clinique (IREC), Cliniques Universitaires Saint Luc, Brussels, Belgium; ^7^Bayer Consumer Care, Basel, Switzerland; ^8^Bayer HealthCare Pharmaceuticals, Whippany, NJ, United States; ^9^Carolina Urologic Research Center, Myrtle Beach, SC, United States

**Keywords:** targeted alpha therapy, radium-223, Lutetium-177-PSMA, metastatic castration-resistant prostate cancer, real-world practice

## Abstract

Radium-223 dichloride (^223^Ra) is an α-emitter approved for the treatment of metastatic castration-resistant prostate cancer (mCRPC) with bone metastases, but without visceral involvement. Despite being a life-prolonging therapy (LPT), ^223^Ra remains underutilized. A large body of real-world evidence (RWE) for ^223^Ra has been published in the decade since the pivotal phase 3 ALSYMPCA study, a period during which the treatment landscape has continued to evolve. How to optimize ^223^Ra use, including how to integrate it into the mCRPC management pathway amongst other current LPTs (i.e., with respect to timing and concurrent, layered, or sequential use), is therefore of considerable interest. RWE studies lack the conventional restraints of clinical trials and can therefore help to build an understanding of how treatments may be best used in routine practice. Here we review RWE studies investigating the efficacy and safety of ^223^Ra in mCRPC [including in sequence with the recently approved 177-Lutetium conjugated to the ligand prostate-specific membrane antigen (^177^Lu-PSMA)], as well as response marker development, imaging techniques, and current clinical practice recommendations.

## 1 Introduction

The radionuclide radium-223 dichloride (^223^Ra) is a life-prolonging therapy (LPT) in oncology (1), paving the way as the first approved α-emitter. ^223^Ra is approved for the treatment of metastatic castration-resistant prostate cancer (mCRPC) with bone metastases, but without visceral involvement (2, 3), with metastatic prostate cancer being primarily a bone-related disease (4), unlike other cancers. This approval was based on improvements in overall survival (OS) vs. placebo [14.9 vs. 11.3 months; hazard ratio (HR) 0.70; 95% confidence interval (CI) 0.58–0.83, *P* < 0.001] in patients with mCRPC (including those with low-volume lymph node metastases), with or without prior chemotherapy, in the pivotal phase 3 ALSYMPCA study (5).

In addition to investigating efficacy and safety in a real-world setting, the challenges of ^223^Ra being the first approved α-emitter (e.g., accessibility and understanding of mechanism of action and appropriate usage) also needed to be overcome, with implementation (logistics) and physician and patient education being key to its uptake in clinical practice. However, ^223^Ra remains underutilized for various reasons, including lack of prostate-specific antigen (PSA) response, intravenous administration issues and the continued use of back-to-back androgen receptor pathway inhibitor (ARPI) regimens [despite a lack of ARPI re-challenge efficacy and current guidelines (6–9) recommending multiple lines of ARPIs are avoided] (10, 11).

Since ALSYMPCA completion, the treatment landscape has evolved. Several currently approved LPTs, specifically the ARPIs abiraterone (12, 13) enzalutamide (14, 15), apalutamide (16), and darolutamide (17), the poly (adenosine diphosphate-ribose) polymerase inhibitor olaparib (18, 19), the immunotherapy sipuleucel-T (20), and 177-Lutetium conjugated to the ligand prostate-specific membrane antigen (^177^Lu-PSMA-617) (21), were unavailable outside of randomized clinical trials (RCTs) during ALSYMPCA. Furthermore, although docetaxel and cabazitaxel were approved in mCRPC at the time of ALSYMPCA, their position in the treatment pathway has since changed. Consequently, ensuring the appropriate choice of patients and treatment sequence for ^223^Ra is key to maximizing therapeutic benefit. There is thus a need for RCTs of ^223^Ra regimens in the current mCRPC landscape, some of which are currently underway [RADIANT (phase 4, ^223^Ra vs. ARPI), PEACE III (phase 3, ^223^Ra plus enzalutamide vs. enzalutamide alone) and DORA (phase 3, ^223^Ra plus docetaxel vs. docetaxel alone)] (22–24), and for real-world evidence (RWE).

Unlike RCTs, RWE gathers data from non-interventional studies, clinical registries and other sources reflecting routine clinical practice, thus helping to refine a treatment’s therapeutic index without conventional RCT constraints (25). RWE studies can complement RCTs, especially for patients ineligible for RCT inclusion and where Level 1 evidence is lacking. Despite recommended treatment algorithms, variability exists in individual treatment pathways, particularly with some mCRPC therapeutic options moving to earlier disease stages and issues around undertreatment (26, 27). Here we review RWE studies (retrospective unless otherwise specified) investigating ^223^Ra in mCRPC, with discussion focusing on studies with *N* > 100, except where data are limited.

## 2 Efficacy

Real-world OS in patients treated with ^223^Ra was 8.2–29 months (Supplementary Table 1), a range that encompasses the median OS of 14.9 months reported in ALSYMPCA. However, survival outcomes are influenced by patient selection as well as therapy choice, and the studies included in this review vary by patient characteristics, study designs, and prior therapies.

### 2.1 Treatment completion

**FIGURE 1:**
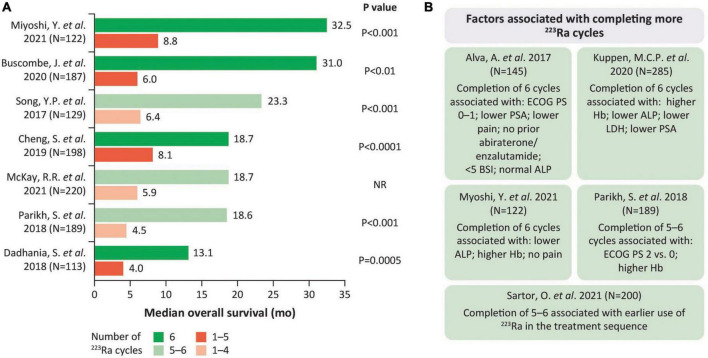
Completing more ^223^Ra cycles is associated with longer OS. **(A)** Median OS by ^223^Ra cycle number. **(B)** Factors associated with completing more ^223^Ra cycles. ALP, alkaline phosphatase; BSI, bone scan index; ECOG PS, Eastern Cooperative Oncology Group performance status; Hb, hemoglobin; LDH, lactate dehydrogenase; OS, overall survival; PSA, prostate-specific antigen.

OS benefits were more notable (*P* < 0.01 where reported) in patients who completed 5–6 vs. fewer cycles of ^223^Ra (28–34) (Figure 1A). Factors associated with completion of 5–6 cycles in some studies included certain patient/disease characteristics (29, 33, 35, 36) [e.g., lower PSA or alkaline phosphatase (ALP) (35) and absolute neutrophil count at least lower limit of normal (36)] and earlier ^223^Ra use (29) (Figure 1B). Indeed, there was a higher likelihood of completing all 6 cycles of ^223^Ra when it was given prior- vs. post-chemotherapy (*P* < 0.001) (32). However, ^223^Ra position in the treatment sequence (i.e., line 1 vs. 2 or ≥ 3) had no impact on treatment completion in another study (35). Moreover, there was also a greater likelihood of the mean number of ^223^Ra cycles being higher when ^223^Ra was used as combination therapy rather than monotherapy (*P* = 0.003) (32).

### 2.2 Treatment sequence

^223^Ra use earlier in the mCRPC treatment pathway may improve survival outcomes, according to some studies (35, 37). Median survival was greater in patients with one vs. two prior therapies (14.7 vs. 11.2 months; *P* = 0.03) in one study (37), and another demonstrated worse OS with ^223^Ra used as line ≥ 3 vs. line 1 (HR 3.267; *P* < 0.01) (35). However, other studies found OS did not significantly differ by prior line of therapy (0 vs. ≥ 1 or across lines 0, 1, 2, 3, or 4) (38), and was generally similar (14.3–14.7 months) when ^223^Ra was given immediately after abiraterone as treatment line 2, 3, or ≥ 4 (39).

Similarly, a greater OS benefit was seen with ^223^Ra used pre- vs. post-chemotherapy in one study (12.3 vs. 8.1 months; *P* = 0.02), although prior enzalutamide or abiraterone plus prednisolone treatment had no significant OS impact (40). By contrast, another study found no significant OS difference with ^223^Ra pre- vs. post chemotherapy (including when patients receiving ^223^Ra in combination with enzalutamide or abiraterone were excluded; the safety of these combinations are discussed in section “3 Safety”) (32). Furthermore, prior cabazitaxel use was not a predictor of OS in a prospective registry analysis (41), and prior docetaxel use had no significant impact on survival in another study (34).

## 3 Safety

In short- and long-term analyses of ALSYMPCA, ^223^Ra had limited myelosuppressive effects and was well tolerated, without major safety concerns (5, 42). RWE has similarly indicated that ^223^Ra is safe and well tolerated in patients with mCRPC (Supplementary Table 2), and importantly demonstrated a lack of rare treatment-emergent adverse events (TEAEs), e.g., second malignancies or cardiovascular events, which RCTs would be underpowered to detect.

When ^223^Ra monotherapy was compared with standard-of-care, the estimated 36-month fracture risk in the respective groups was 19% vs. 10% (HR 1.61; 95% CI: 0.96–3.02) (43). Regimens combining use of ^223^Ra and abiraterone (plus prednisolone) or enzalutamide have been reported in real-world studies (44–48). However, based on a significantly increased risk of fractures when ^223^Ra was used in combination with abiraterone plus prednisolone in the ERA 223 phase 3 RCT (49), this combination is now contraindicated in the EU (2) and is not recommended in the US (3). Of note, in the ERA 223 trial, the incidence of fractures was lower in patients who were taking bone protecting agents (bisphosphonates or denosumab) at baseline (15 and 7% in the ^223^Ra and placebo groups, respectively) than in patients not taking bone protective agents (37 and 15%, respectively) (49). Furthermore, an increased fracture risk was also reported with ^223^Ra plus enzalutamide vs. enzalutamide in the phase 3 PEACE III RCT, although fracture risk was largely eliminated in each treatment group with preventative use of bone protecting agents (denosumab and zoledronic acid) (50). Increased fracture risk due to therapy-induced bone loss has been seen for several systemic therapies for prostate cancer, and fracture risk is increased in patients with bone metastases (51). As such, the importance of regularly evaluating bone health and the use of bone protective agents in patients with prostate cancer has been highlighted in the recommendations of a working group of European experts (51).

### 3.1 Treatment sequence

RWE suggests that ^223^Ra is generally well tolerated, irrespective of prior chemotherapy status, although prior chemotherapy may be associated with an increased likelihood of hematological events (52, 53), possibly due to patients having more advanced disease (e.g., bone marrow involvement) and/or prior chemotherapy toxicities.

For example, in the first interim analysis of the REASSURE study, prior chemotherapy status generally did not affect the overall safety profile of ^223^Ra, with the incidence of drug-related TEAEs being 41 and 36% with or without prior chemotherapy (53). However, drug-related hematologic TEAEs were more than twice as frequent in patients with than without prior chemotherapy (21% vs. 9%) (53). Moreover, in a prospective Japanese study, although there was no marked difference between patients with or without prior chemotherapy with regard to the incidence of drug-related TEAEs (29% vs. 25%), including hematological TEAEs (18% vs. 17%), with ^223^Ra, the incidence of both events was notably numerically greater in patients who had received two lines of prior chemotherapy (36 and 24%) (52).

Furthermore, the CAPRI registry found a significant (*P* ≤ 0.015) increase in the incidence of grade ≥ 2 anemia, grade ≥ 2 thrombocytopenia and blood transfusions with later-line use of ^223^Ra (line ≥ 3 vs. 2 vs. 1), although symptomatic skeletal event (SSE) incidence was not impacted (35). Factors associated with grade ≥ 2 hematological abnormalities include low hemoglobin (Hb) and low platelet count at baseline (52). Of note, ^223^Ra requires patient hematological evaluation before every dose and caution (2)/close monitoring (3) is advised for patients with evidence of compromised bone marrow reserve.

In an assessment of fracture risk by line of therapy, the estimated adjusted 36-month fracture risk with ^223^Ra vs. standard-of-care was 18% vs. 12% (HR 1.14; 95% CI, 0.50–2.15) when first line and 16% vs. 9% (HR 1.86; 95% CI: 0.62–10.93) when second line. Later treatment lines had too few fractures for analysis (43).

## 4 ^223^Ra therapy/^177^Lu-PSMA treatment sequence and interval duration

^177^Lu-PSMA targets prostate cancer *via* a different mechanism to ^223^Ra. ^177^Lu-PSMA delivers β-particle radiation to PSMA-expressing tumor cells. In the VISION RCT, ^177^Lu-PSMA-617 plus standard-of-care prolonged OS vs. standard-of-care alone (15.3 vs. 11.3 months; HR for death 0.62; *P* < 0.001) (21). Among the 17.4% of patients who had previously received ^223^Ra, ^177^Lu-PSMA-617 efficacy was not adversely affected (54), although safety has not been reported for these patients.

Although limited by small patient numbers, real-world studies have demonstrated the clinical feasibility of giving ^177^Lu-PSMA after ^223^Ra and indicate this treatment sequence has an acceptable safety profile (55–58). In a *post hoc* analysis of REASSURE, median OS from start of ^177^Lu-PSMA was 13.2 months in patients who had previously received ^223^Ra (56). Moreover, in a large retrospective study, median OS was not significantly different in patients who did vs. did not receive prior ^223^Ra (10.8 vs. 11.3 months) (55). Furthermore, interim analyses of the RALU study, which investigated ^177^Lu-PSMA use in patients previously treated with ^223^Ra, found this approach to be clinically feasible (median OS 12.6 months; 95% CI: 8.8–16.1) and well tolerated (58).

Another consideration around treatment sequencing with radionuclide therapies is the treatment interval. Early initiation of ^177^Lu-PSMA within 8 weeks of ^223^Ra treatment (during which disease progression had occurred) was effective and did not reveal major safety concerns (57).

Thus, sequential treatment with ^223^Ra and ^177^Lu-PSMA is feasible and can be factored into considerations around optimal sequencing of the LPTs available for patients with mCRPC. However, further studies are warranted.

## 5 Development of response markers

Surrogate markers predicting treatment outcomes with ^223^Ra are needed to monitor and achieve optimal treatment duration and to identify patient subpopulations who may benefit most from ^223^Ra. Multiple RWE studies have investigated potential markers of survival (Supplementary Table 3), with this section focusing on multivariate analyses.

### 5.1 Laboratory parameters

Multivariate analyses have found various factors to be associated with survival outcomes. Baseline Hb was found to be prognostic of OS (59) and elevated baseline Hb (≥ 120 g/L) was associated with increased OS (60), whereas low baseline albumin (< 35 g/L) (61) and elevated PSA (> 80 μg/L) (61) were associated with poor OS. Similarly, other factors prognostic of OS include baseline neutrophil-to-lymphocyte ratio (28), baseline lactose dehydrogenase (62) [with elevated lactose dehydrogenase associated with shorter OS (41)] and higher baseline ALP (28) [with ALP > 150 U/L associated with poor OS (61)]. Elevated baseline ALP without a subsequent ALP decline of ≥ 10% following the first ^223^Ra dose was also prognostic of shorter OS (62).

### 5.2 Clinical parameters

A number of clinical parameters have been associated with patient survival. In terms of patient demographics, age was found to be a predictor of OS (28), with an age of > 75 years being associated with reduced OS (63). Moreover, in an analysis of US electronic heath records of mainly Caucasian patients (73.5%), other race (Asian, Hispanic, Latino, or other) was associated with improved survival (63). With regard to disease characteristics, visceral metastases (63) and prior SSEs (63) reduced OS, whereas bone-only metastases were associated with longer OS (41). Eastern Cooperative Oncology Group performance status (ECOG PS) was also prognostic of OS (59, 62), with ECOG PS 2–3 (61) and ECOG PS 2–4 (63) associated with worse OS and ECOG PS 0–1 associated with increased OS (60). Another clinical parameter prognostic of OS was number of prior systemic therapies (62). Prior chemotherapy use reduced OS (63), whereas no prior use of docetaxel increased OS (60). As discussed in section “2.1 Treatment completion,” the number of completed cycles of ^223^Ra (5–6 vs. 1–4) was also a predictor of OS (28).

### 5.3 Composite markers

Several studies have reported composite prognostic scoring methods aimed at identifying patients that may benefit most from ^223^Ra therapy (59–61, 64). A composite score derived from combining baseline Hb ≥ 120 g/L, total ALP ≤ 110 U/L and ECOG PS 0–1 identified patients with a low-, intermediate- or high-risk of death (composite score 2, 3–4 and 5–6, respectively; median OS 23, 8, and 5 months) (60). A similar 3-variable prognostic score combining baseline ECOG PS, Hb < 12 g/dL and PSA ≥ 20 ng/mL was predictive of OS in an initial cohort (64), with subsequent validation in a larger cohort (59). In the larger cohort, patients in the low (score 0), moderate (score 1–2), or high (score 3–4)-risk groups had a median OS of 33, 16, and 8 months, respectively (59). Likewise, a scoring system that combined albumin < 35 g/L, ALP > 150 U/L, PSA > 80 μg/L, and ECOG PS 2–3 identified three patient groups with different OS outcomes, namely good (score 0–1; median OS 19.4 months), intermediate (score 2; median OS 10.0 months) and poor (score 3–4; median OS 3.1 months) (61).

## 6 Imaging

An expert consensus developed at the European Association of Nuclear Medicine Focus 1 meeting concluded that, for patients with mCRPC who are candidates for ^223^Ra, bone scintigraphy is the recommended pre-treatment imaging method. Consensus was not reached as to which imaging method should be used for monitoring treatment response, although bone scintigraphy was favored by most (14/21) panelists (65).

Automated bone scan index (BSI) is useful for assessing skeletal metastases. Baseline BSI was associated with OS in patients who received ^223^Ra in two studies (66, 67), with median OS being 8.2 and 15.0 months in patients with BSIs of > 5 or ≤ 5, respectively (HR 2.65; 95% CI: 1.5–4.7; *P* = 0.001) (67). However, only one of the two studies found a significant association between on-treatment BSI and OS (66). A potential limitation of this approach is the potential uptake of bone scintigraphy agents into healing bone which could confound results (66).

Radionuclide cancer therapies offer considerable potential for personalized treatment as their physical properties enable *in vivo* imaging of their uptake and retention (68). ^223^Ra administration is *via* body weight-adjusted standard dosing regimens, although patient-specific dosimetry and treatment optimization may be possible *via* quantitative imaging with ^223^Ra (68). Although ^223^Ra imaging showed intra- and inter-patient variability for ^223^Ra dose absorption in metastases, there was a relationship between lesion-absorbed dose and treatment response (69). ^18^F-fluoride, like ^223^Ra, localizes primarily to areas of osteoblastic activity in bone and has potential as a surrogate measure of the absorbed ^223^Ra dose (69). ^18^F-fluoride uptake into bone metastases correlated significantly with that of ^223^Ra, as well as the absorbed ^223^Ra dose and resultant response (69).

Notably, PSMA-positron emission tomography (PET) has been shown to be more sensitive than bone scintigraphy in detecting bone metastases in patients with prostate cancer (70). High PSMA expression on planar/single-photon emission computed tomography (SPECT) or PET/CT scans following standard therapies for mCRPC, including ^223^Ra, was associated with worse OS than low PSMA expression (71).

## 7 Clinical practice recommendations

^223^Ra is recommended for mCRPC in all major treatment guidelines (6–9) and has the highest possible clinical benefit score for non-curative therapies in mCRPC in the ESMO-Magnitude of Clinical Benefit Scale (indicating a substantial magnitude of clinical benefit) (72). Expert recommendations from 11 nuclear medicine centers across six European countries provide additional insights on how to optimize ^223^Ra use (73). These include guidance for center organization/preparation, ^223^Ra ordering, preparation and disposal, ^223^Ra treatment delivery/administration, and patient referral/experience, and highlight the importance of starting ^223^Ra treatment as soon as possible in eligible patients (including those with early symptoms of bone metastases) (73).

However, for ^223^Ra to meet the inherent complex needs of patients, communication and coordination within multidisciplinary teams (i.e., nuclear medicine, oncology, and urology services) and centers is advised (73). Communication between the nuclear medicine physician and other specialties is important to maintain awareness for whom and when ^223^Ra may be appropriate, and to inform of developments in prostate cancer management (including nuclear medicine options) (73). With regard to such developments, when the Advanced Prostate Cancer Consensus Conference discussed questions relating to ^223^Ra and other therapies in 2021, consensus was reached that using ^223^Ra after ^177^Lu-PSMA is safe (76% consensus), based on outcomes from VISION, in which approximately 2.5% of patients received ^223^Ra following ^177^Lu-PSMA therapy (74). RWE supporting use of ^223^Ra followed by ^177^Lu-PSMA are discussed in section “4 ^223^Ra therapy/^177^Lu-PSMA treatment sequence and interval duration.”

## 8 Discussion

For patients with mCRPC, it is important to offer as many approved LPTs as possible. Real-world studies can help healthcare professionals understand how best to utilize currently available treatment options, such as ^223^Ra, and are used by regulatory bodies in decision making (75–78). Although there are well recognized limitations to these studies, including confounding factors, various types of bias (pertaining to selection, patient/caregiver recall, event detection, and data misclassification) and missing data (limiting statistical power), they can complement/supplement clinical trial data and help to determine whether RCT evidence is generalizable to patient populations in clinical practice (79, 80).

The large body of RWE that has emerged for ^223^Ra in recent years indicates that ^223^Ra is an effective and safe LPT option in mCRPC, supporting RCT findings. Completing 5–6 ^223^Ra cycles was associated with better survival outcomes across real-world studies, highlighting the value of being able to identify patients most capable of completing therapy. RWE indicates several potential markers that may help to do this, although these are not yet validated in prospective studies. A potential challenge in optimizing ^223^Ra use in clinical practice is how to best integrate it into the mCRPC treatment pathway. However, as current RWE has been variable in this regard, there is a need to further evaluate ^223^Ra in the context of other treatments with respect to timing and concurrent, layered, or sequential use, and the effectiveness and safety of such treatment approaches. To this end, several clinical trials (e.g., PEACE-III; AlphaBet; COMRADE; Rad2Nivo; RADIANT; DORA) (22–24, 81–83) and RWE studies (e.g., REASSURE; RaLu) (58, 84) continue to explore ^223^Ra use in mCRPC.

## Author contributions

JO’S, RM, KR, KF, DG, BT, AS, PS, FV, and NS contributed to the conception and design, drafting and revising of the work, and approval of the final version. All authors agreed to be accountable for all aspects of the respective work.
