# Supplementary material for: Real-world effectiveness, long-term safety and treatment pathway integration of radium-223 therapy in patients with metastatic castration-resistant prostate cancer
Source: Front Med (Lausanne). 2022 Dec 22;9:fmed-09-1070392. doi: 10.3389/fmed.2022.1070392 (PMC9812947; doi:10.3389/fmed.2022.1070392)
Supplement: Supplementary file 1 [file Table_1.DOCX]

**Supplementary information for “Real-world Effectiveness, Long-Term Safety and Treatment Pathway Integration of Radium-223 Therapy in Patients with Metastatic Castration-Resistant Prostate Cancer”**

**Joe M. O’Sullivan, Rana R. McKay, Kambiz Rahbar, Karim Fizazi, Daniel J. George, Bertrand Tombal, Anja Schmall, Per Sandström, Frank Verholen, Neal Shore**

**Supplementary Table 1.** Effectiveness of ^223^Ra in real-world studies

| - **Reference** | - **Country** | - **Number of pts** | - **Study design** | - **Outcomes including survival** |
| --- | --- | --- | --- | --- |
| Weng WC, et al. Ra-223 for metastatic, castration-resistant prostate cancer: A retrospective chart review study of real-world use in a tertiary hospital in Taiwan. *J Formos Med Assoc.* 2022;2:S0929-6646(22)00040-7 | - Taiwan | - 36 | - Retrospective chart review, - single center | - ^223^Ra as first-line therapy n=12, second-line therapy n=11, third-line therapy n=13 - Significantly decreased pain scores with second- and third-line ^223^Ra - PSA significantly increased from baseline in patients treated with third-line ^223^Ra and ^223^Ra post-chemotherapy - ALP level significantly decreased from baseline in the setting naïve for novel anti-hormone therapy and chemotherapy |
| - Hyväkkä A, et al. Radium-223 dichloride treatment in metastatic castration-resistant prostate cancer in Finland: A real-world evidence multicenter study. *Cancer Med*. 2022; 00:1–13. doi: 10.1002/cam4.5262 | - Finland | - 160 | - Retrospective, multicenter | - Median OS 13.8 months (range 0.5–57 months) - Median PFS 4.9 months (range 0.5–29.8 months) - ALP values within normal range before and during ^223^Ra treatment, or reduction of elevated ALP to normal during treatment, were associated with improved OS versus elevated ALP values before and during treatment (P<0.0001). - PSA ≥100 μg/L prior to 223Ra was associated with worse OS versus low PSA (<20 μg/L) (P=0.0001) - 57% of patients experienced pain relief, and pain relief was associated with better OS (P=0.002) |
| - Sartor O, et al. Real-world outcomes of second novel hormonal therapy or radium-223 following first novel hormonal therapy for mCRPC. *Future Oncol.* 2022;18(1):35-45 | - USA | - 346 | - Retrospective analysis of the US Flatiron database | - In the ^223^Ra arm (n=120) proportionally more pts had prior SSEs and bone-only metastases than in the alternative NHT arm (n=226), and first-line NHT duration was shorter in the ^223^Ra arm - Following second-line therapy, 49% of pts received subsequent life-prolonging therapy in the ^223^Ra vs 39% of pts in the NHT arm; of these, 47% vs 76% received taxane - Median OS was 10.8 m in the ^223^Ra arm and 11.2 m in the NHT arm |
| - Kim SE, et al. A real-world evaluation of radium-223 in combination with abiraterone or enzalutamide for the treatment of metastatic castration-resistant prostate cancer. PLoS One. 2021;16(6):e0253021 | - USA | - 60 | - Retrospective cohort study using electronic health record data, - single center | - 41 pts received ^223^Ra monotherapy and 19 the combination of ^223^Ra and either abiraterone or enzalutamide (combination arm) - Median OS: 12.7 m in the ^223^Ra arm vs 12.8 m in the combination arm (HR 1.15, 95% CI 0.59-2.23; P = 0.68) - Median PFS: 7.6 m in the ^223^Ra vs 4.9 m in the combination arm (HR 1.94, 95% CI 1.11-3.40; P = 0.02) - No significant differences in time to first SSE (P = 0.97), SSE-free survival (P=0.16), or in the overall incidence of SAEs (P = 0.45) |
| - Sartor O, et al. Clinical outcomes, management, and treatment patterns in patients with metastatic castration-resistant prostate cancer treated with radium-223 in community compared to academic settings. *Prostate.* 2021 Jul;81(10):657-666 | - USA | - 200 | - Retrospective | - 57% of pts were treated in CPs - The period from diagnosis of mCRPC to treatment was 1.3 years in CPs and 1.9 years in ACs (P < 0.001); pts in CPs received a greater mean number of ^223^Ra doses (5.4 vs 4.8; P = 0.001) - OS was 21.6 m in CPs and 20.7 m in ACs (P = 0.306) - Pts who received 5-6 ^233^Ra doses had a longer median OS than those with 1-4 doses (23.3 vs 6.4 m; P < 0.001) - 43% of pts received ^223^Ra monotherapy and 57% ^223^Ra plus other agents - Most pts received ^223^Ra concurrently with abiraterone acetate or enzalutamide - Most pts could complete 5-6 doses of ^223^Ra |
| Arays R, et al. Review of palliative (223)Ra in metastatic castration-resistant prostate cancer: Experience at West Virginia University Cancer Center. *J Nucl Med Technol.* 2021;49(1):70-74. | - USA | - 24 | Retrospective, observational | - All pts received ADT and 79% docetaxel prior ^223^Ra - 79% received 33 courses of radiation - 46% received 6 ^223^Ra doses - Median OS from the first ^223^Ra infusion to the last follow-up or death was 8.3 m (range 0-44): this was nearly 50% shorter than the ALSYMPCA OS of 14.9 m (P = 0.01) - Optimal clinical benefit may require earlier referral for ^223^Ra |
| - Frantellizzi V, et al. A national multicenter study on overall survival in elderly metastatic castrate-resistant prostate cancer patients treated with radium-223. *Aging Clin Exp Res.* 2021;33(3):651-658 | - Italy | - 430 | - Retrospective, multicenter | - 47% of the pts were < 75 years old and 53% were ≥ 75 years old - OS did not show significant differences in both groups |
| Kuppen MC, et al. Real-world outcomes of radium-223 dichloride for metastatic castration resistant prostate cancer. *Future Oncol.* 2020 Jul;16(19):1371-1384 | - The Netherlands | - 285 | - Retrospective using the CAPRI registry | - 49% received ^223^Ra in ≥ 3 therapy line - 51% completed 6 injections - ^223^Ra in ≥ 3 therapy line was associated with worse OS (HR: 3.267; 95% CI: 1.689-6.317; P < 0.01) - 34% had an SSE after first ^223^Ra cycle without differences between subgroups |
| Jiang XY, et al. Optimising radium 223 therapy for metastatic castration-resistant prostate cancer-5-year real-world outcome: Focusing on treatment sequence and quality of life. *Clin Oncol (R Coll Radiol).* 2020;32(10):e177-e187 | - UK | - 228 | Prospective   - service evaluation, single center | - Medium OS was 11.1 m - In post-chemotherapy vs chemotherapy-naïve group, OS was 8.1 vs 12.3 m (HR 1.52, 95% CI 1.06-2.17, P = 0.02) - In pre-enzalutamide vs post-enzalutamide group, 11.3 vs 10.4 m (HR 0.92, 95% CI 0.63-1.33, P = 0.65) - In pre-abiraterone/prednisolone vs post-abiraterone/prednisolone arm, 11.8 vs 10.5 m (HR 0.74, 95% CI 0.51-1.06, P = 0.08); in this latter group, the fracture rate was 24% (15/63) - Pain scores post-^223^Ra (n = 101) improved in 54%, remained unchanged in 17% and worsened in 30% of pts - Overall QoL scores showed a similar trend - QoL was not significantly associated with OS |
| Shore N, et al. Concurrent or layered treatment with radium-223 and enzalutamide or abiraterone/prednisone: real-world clinical outcomes in patients with metastatic castration-resistant prostate cancer. *Prostate Cancer Prostatic Dis.* 2020;23(4):680-688 | - USA, Canada, Belgium, Germany | - 625 | - Retrospective using electronic health record data from the Flatiron Health database | - 22% of pts received ^223^Ra together with abiraterone/prednisone and 27% with enzalutamide - 73% of pts layered, 23% concurrent - Median OS from mCRPC diagnosis was 28.1 months |
| Cheng S, et al. Population-based analysis of the use of radium-223 for bone-metastatic castration-resistant prostate cancer In Ontario, and of factors associated with treatment completion and outcome. *Cancer Manag Res.* 2019;11:9307-9319 | - Canada | - 198 | - Retrospective chart analysis | - The ^223^Ra completion rate was 46.5% - 92.4% of pts were pre-treated with abiraterone and/or enzalutamide, and 48.5% of these pts received docetaxel - Actuarial median survival was 13.3 m - Pts with incomplete ^223^Ra had median survival of 8.1 m (6.0-12.2) and with complete ^223^Ra 18.7 m (15.3-22.3) (P < 0.0001) - Early ^223^Ra discontinuation, baseline anemia, high PSA, prior SREs, visceral metastases, and being referred to another centre for ^223^Ra therapy were associated with worse outcome |
| Boni G, et al. (223)Ra-chloride therapy in men with hormone-refractory prostate cancer and skeletal metastases: Real-world experience. *Tumori.* 2018;104(2):128-136 | - Italy | - 83 | - Retrospective, observational | - 41 pts completed 6 treatment cycles - NRS pain scores significantly improved at end of treatment (P < 0.000001) - OS was a mean of 10.1 m (median OS not reached) - Kaplan-Meier OS and PFS estimates were 17.5 m and 7.7 m, respectively - OS and PFS significantly correlated with number of ^223^Ra cycles |
| Minekawa TB, et al. Single center developing country analysis of radium-223 therapy in prostate cancer-preliminary results. *Am J Nucl Med Mol* *Imaging*. 2021;11(5):352-362 | - Brazil | - 28 | - Retrospective chart review, single center | - Overall median OS was 15.6 m (9.1 m with ≤ 4 cycles, 18.5 m with 5 or 6 cycles) - 76.2% of pts who completed 6 cycles did not present bone events, while 71.4% who had SREs completed < 6 cycles - 82.1% of pts received concomitant therapies with no significant side effects |
| Badrising SK, et al. Integrated analysis of pain, health-related quality of life, and analgesic use in patients with metastatic castration-resistant prostate cancer treated with radium-223. *Prostate Cancer Prostatic Dis*. 2022; 25:248–255 doi: 10.1038/s41391-021-00412-6. | - The Netherlands | - 300 | - Retrospective | - Complete pain response was achieved in 31.4% of pts - The median TTP and TTFD were 5.6 and 5.7 m, respectively |
| Buscombe J, et al. Quantifying the survival benefit of completing all the six cycles of radium-223 therapy in patients with castrate-resistant prostate cancer with predominant bone metastases. *World J Nucl Med.* 2020;20(2):139-144 | - UK | - 187 | - Retrospective, single center | - Median OS: All patients, 15 m (patients receiving 6 cycles of ^223^Ra (n=107), 31 m; patients receiving <6 cycles (n=80), 6 m [P = 0.001]) - 1-year survival rate was 87% with 6 cycles and 30% for < 6 cycles |
| Klain M, et al. Combined bone scintigraphy and fluorocholine PET/computed tomography predicts  response to radium-223 therapy in patients with prostate cancer. *Future Sci OA*. 2021;7(8):FSO719 | - Italy | - 48 | - Retrospective, multicenter | - Pain relief was observed in 27 pts - Pts with more lesions at 18F-fluorocholine PET/CT than at bone scintigraphy had a poorer prognosis |
| Caffo O, et al. Sequencing life-prolonging agents in castration-resistant prostate cancer patients: Comparison of sequences with and without (223)Ra. *Cancer Biother Radiopharm.* 2021;36(5):391-396 | - Italy | - 264 | - Retrospective chart review | - Pts were sequentially treated with two or three life-prolonging therapies, with or without ^223^Ra, after first-line docetaxel - Median cumulative OS from the start of first-line therapy was 40.6 m in the ^223^Ra group (n = 78) and 36.2 m in the non-^223^Ra group (n=186) (P = 0.08) |
| Ahmed M, et al. Radium-223 in the third-line setting in metastatic castration-resistant prostate cancer: Impact of concomitant use of enzalutamide on overall survival (OS) and predictors of improved OS. *Clin Genitourin Cancer.* 2021 Jun;19(3):223-229 | - US | - 51 | - Retrospective, single center | - ^223^Ra in the third-line setting with or without enzalutamide was investigated - Median OS was 20.4 m for ^223^Ra and 17.5 m for ^223^Ra plus enzalutamide (P = 0.5186) |
| Caffo O, et al. Sequencing radium 223 and other life-prolonging agents in castration-resistant prostate cancer patients. *Future Oncol.* 2021;17(7):807-815 | - Italy | - NR | - Retrospective, multicenter | - Review of clinical records of pts who had received at least three LPs, including ^223^Ra - Median OS from the start of first-line treatment was 39.8 m, with pts who completed 6 cycles of ^223^Ra having a longer OS than those who did not (53.2 vs 29.5 m; P < 0.0001) - Activity of ^223^Ra confirmed regardless of treatment line |
| Jarvis P & Sundram F. Radium-223 therapy for metastatic castration-resistant prostate cancer: survival benefit when used earlier in the treatment pathway. *Nucl Med Commun*. 2021;42(3):332-336 | - UK | - 191 | - Retrospective, single center | - 63% of pts received one prior therapy before ^223^Ra (group A), 37% received two prior therapies (group B) - Median survival in group A was significantly improved, compared with group B (448 days vs 341 days; P = 0.03) - Survival was improved if all 6 cycles of ^223^Ra were completed |
| Arays R, et al. Review of Palliative (223)Ra in Metastatic Castration-Resistant Prostate Cancer: Experience at West Virginia University Cancer Center. *J Nucl Med Technol.* 2021;49(1):70-74 | - US | - 24 | - Retrospective, single center | - All pts received prior androgen deprivation therapy; 79% received docetaxel and 79% radiation - 46% completed all 6 cycles of ^223^Ra - Median OS from first ^223^Ra infusion was 8.3 m |
| Sraieb M, et al. Assessing the quality of life of patients with metastatic castration-resistant prostate cancer with bone metastases receiving [223Ra]RaCl2 therapy*. Medicine (Baltimore)*. 2020;99(38):e22287 | - Germany | - 30 | - Prospective | - EORTC questionnaire was given before treatment, after first, third and fifth cycle and end of treatment - Median OS was 26 m - Compared with baseline, only the scale "role functioning" showed a temporary worsening after the first therapy cycle (P = 0.03) - In subsequent cycles, its mean value rose to initial levels - All other functional and symptom scales, as well as global health status, remained constant over all time points and showed no significant changes (P > 0 .05) |
| Hashimoto K, et al. Dynamic changes of bone metastasis predict bone predominant status to benefit from radium-223 dichloride for patients with castration-resistant prostate cancer. *Cancer Med*. 2020;9(22):8579-8588 | - Japan | - 127 | - Retrospective, multicenter | - Pts were divided into groups based on changes in bone lesions between diagnosis and ^223^Ra treatment: A – only known lesions, B – *de novo* lesions, C – new progressive lesions - Median PFS was 11.3 m in group A, 8.1 m in B and 5.1 m in C (P < 0.001) |
| Yamamoto Y, et al. Clinical indicators for predicting prognosis after radium-223 administration in castration-resistant prostate cancer with bone metastases. *Int J Clin Oncol.* 2021;26(1):192-198 | - Japan | - 42 | - Retrospective chart review, single center | - Two thirds of patients had received at least two lines of previous therapy - Median OS was 16.6 m |
| McKay R, et al. Treatment of metastatic castration resistant prostate cancer with radium-223: a retrospective study at a US tertiary oncology center. *Prostate Cancer Prostatic Dis*. 2021;24(1):210-219 | - US | - 220 | - Retrospective, single center | - Mean line of mCRPC therapy of ^223^Ra was 3rd and 5th when given pre- and post-chemotherapy, respectively - Median OS from first mCRPC treatment was 39.4 m (95% CI: 33.0-48.8) for pts with ^223^Ra pre-chemotherapy vs 37.4 m (95% CI 32.0, 43.5) post-chemotherapy - Median OS was 35.2 m (95% CI 27.9-43.3) vs 32.0 m (95% CI 26.9-36.0) for pts with ^223^Ra combination vs monotherapy |
| Rizzini EL, et al. Clinical aspects of mCRPC management in patients treated with radium-223. *Sci Rep.* 2020;10(1):6681 | - Italy | - 63 | - Retrospective | - ^223^Ra was administered as first-line therapy in 11 pts, as second-line in 19 pts, as third-line in 16 pts and in successive lines in 17 pts - 67% of pts completed 6 ^223^Ra cycles - Median OS was 15 m and median PFS was 8 m |
| Kapoor A, et al. Single-center experience with radium-223 in patients with castration-resistant prostate cancer and bone metastases. *Asian J Androl.* 2020;22(4):437-438. | - USA | - 60 | - Retrospective | - The median OS was 8.2 m, regardless of previous therapy (abiraterone, enzalutamide, docetaxel) - 55% of pts had a reduction in ALP (> 25%) - Time to > 50% increase in ALP from nadir following treatment was 5.5 m and time to PSA rise was 2.1 m |
| Raimondi A, et al. Safety and activity of radium-223 in metastatic castration-resistant prostate cancer: the experience of Istituto Nazionale dei Tumori. *Tumori*. 2020;106(5):406-412 | - Italy | - 41 | - Retrospective, observational | - Pts received a median of 5 cycles (IQR 3-6) - After treatment, 66%, 2%, and 32% of pts had a stable, improved, or deteriorated ECOG PS, respectively - 24%, 61%, and 15% reported a stable, improved, or worsened pain symptom control - Post-treatment ALP was reduced or stable in 46% and PSA in 83% of pts |
| Caffo O, et al. Fracture risk and survival outcomes in metastatic castration-resistant prostate cancer patients sequentially treated with abiraterone acetate and RADIUM-223. Eur J Nucl Med Mol Imaging. 2020;47(11):2633-2638 | - Italy | - 94 | - Retrospective | - 85.1% of pts received ^223^Ra as second- or third-line treatment - Median OS from was >14 months regardless of the ^223^Ra treatment line |
| Parimi S, et al. Pain response in a population-based study of radium-223 (Ra223) for metastatic castration-resistant prostate cancer. *Can Urol Assoc J.* 2019;13(10):E311-E316 | - Canada | - 44 | - Population-based, - single center | - 52% of pts had a pain response - 59% of pts had ALP response >30% - ALP response was seen in 56% of pain-responders vs 43% of non-pain-responders - There was no association between pain response and ALP response (Phi = -0.05; P = 0.77) |
| Prelai A, et al. Radium-223 in patients with metastatic castration-resistant prostate cancer: Efficacy and safety in clinical practice. *Oncol Lett*. 2019;17(2):1467-1476 | - Italy | - 32 | - Retrospective, multicenter | - At scintigraphic assessment: DCR rate of 91%, with 41% partial response; 56% had ALP response and 25% had PSA response; 41% had pain reduction with pain control of 72% - Median PFS was 12 m and median OS 14 m - SRE occurred in three patients and median time to first SRE was 9.5 months |
| Yap K, et al. Impact of timing of administration of bone supportive therapy on pain palliation from radium-223. *Cancer Treat Res Commun.* 2019;18:100114 | - US | - 65 | - Retrospective | - Median number of ^223^Ra doses was 5 - Pain response occurred in 6/6 (100%) pts who received BST within 1 month prior to first ^223^Ra dose and 4/8 (50%) pts who did not receive BST - Pain flare occurred in 29% of pts without BST and 15% with BST (P = 0.44) |
| Dadhania S, et al. Single-centre Experience of use of Radium 223 with clinical outcomes based on number of cycles and bone marrow toxicity. *Anticancer Res.* 2018;38(9):5423-5427 | - UK | - 113 | - Retrospective | - 75% of pts received ^223^Ra prior to docetaxel chemotherapy and 25% after receiving docetaxel - In pts who completed < 6 cycles of ^223^Ra OS was 121 days vs 398 days in those who received 6 cycles (P = 0.0005) |
| Kairemo K, et al. Final outcome of 223Ra-therapy and the role of 18f-fluoride-PET in response evaluation in metastatic castration-resistant prostate cancer-A single institution experience. *Curr Radiopharm*. 2018;11(2):147-152 | - US | - 161 | - Retrospective, single center | - Median OS was 12.4 m |
| Zhang I, et al. Clinical response to radium-223 dichloride in men with metastatic castrate-resistant prostate cancer. *Pract Radiat Oncol*. 2018;8(6):452-457 | - US | - 48 | - Retrospective chart review | - 56% of pts received 6 ^223^Ra cycles - Median OS from first treatment was 16 m - 33% of pts experienced at least one SSE during or after treatment - 62.5% of pts reported a decrease in pain from pre-treatment baseline |
| De Luca R, et al. The clinical efficacy of radium-223 for bone metastasis in patients with castration-resistant prostate cancer: An Italian clinical experience. *Oncology.* 2018;94(3):161-166 | - Italy | - 48 | - Retrospective | - A considerable difference in serum ALP levels before and after treatment was observed, with a significant correlation between pain relief and QoL (P = 0.0021) |
| Wong W, et al. Factors associated with survival following Radium-223 treatment for metastatic castration-resistant prostate cancer. *Clin Genitourin Cancer.* 2017;15(6):e969-e975 | - US | - 64 | - Retrospective, single center | - Median OS was 12.9 m - 33% of pts developed a skeletal event - Median time to first skeletal event was 4.4 m |
| Keizman D, et al. Imaging response during therapy with radium-223 for castration-resistant prostate cancer with bone metastases-analysis of an international multicenter database. Prostate Cancer Prostatic Dis. 2017;20(3):289-293 | - Denmark, Israel, Switzerland | - 130 | Retrospective, multicenter | - 54% of pts completed 6 ^223^Ra injections - 27% of pts showed transient increase in bone metastases-related pain and an improvement of bone metastases-related pain on treatment with ^223^Ra was reported in 49% of pts - At 3 and 6 m of treatment one imaging showed stable disease in 74% and 94% of pts (n = 93/99), respectively - 26% of pts had an increase in the number of bone lesions compared with baseline at 3 months and 6% at 6 m |
| Kuronva Z, et al. A retrospective analysis of the first 41 mCRPC patients with bone pain treated with Radium-223 at the National Institute of Oncology in Hungary. *Pathol Oncol Res.* 2017;23(4):777-783. | - Hungary | - 41 | Retrospective | - 24 patients received ^223^Ra as first-line treatment (58%), 7 patients as second (17%), 3 as third (7.3%), 6 as fourth (14.6%) and 1 as fifth-line therapy (2.4%) - The majority of patients experienced a decrease (37%) or complete cessation (43%) of bone pain intensity |
| Alva A, et al. Clinical correlates of benefit from radium-223 therapy in metastatic castration resistant prostate cancer. *Prostate.* 2017;77(5):479-488 | - USA, Sweden | - 145 | Retrospective | - 51% of pts completed 6 cycles of ^223^Ra - One-year survival was 64% - Survival was highly associated with 6 doses of ^223^Ra - Six ^223^Ra doses were associated with ECOG PS of 0–1, lower baseline PSA and pain level, no prior abiraterone/enzalutamide, < 5 BSI value, and normal ALP - Pain declined in 51% of pts after one dose in pts with baseline bone pain - PSA declined ≥ 50% in 16% of pts - ALP declined ≥ 25% in 48% and ≥50% in 23% of pts |
| Dan TD, et al. Hematologic toxicity of concurrent administration of radium-223 and next-generation antiandrogen therapies. *Am J Clin Oncol.* 2017;40(4):342-347 | - USA | - 25 | Retrospective | - PSA was stable/decreased in 22% of pts receiving ^223^Ra alone vs 35% of pts receiving combination treatment (P = 0.24) |

ACs, academic centers; ADT, androgen deprivation therapy; AEs, adverse events; AIR, annual incidence rate; ALP, alkaline phosphatase; ARATs, androgen-receptor axis-targeted agents; ART, androgen-receptor targeting therapy; BAP, bone-type alkaline phosphatase; BHA, bone health agent; BMI, body mass index; BSI, bone scan index; BST, bone supportive therapy; CAPRI, Castration-resistant Prostate Cancer Registry; CI, confidence interval; CPs, community practices; CRPC, castration-resistant prostate cancer; CT, computed tomography; 1-CTP, serum marker for bone metastasis; DDR, DNA damage repair; EBRT, external beam radiation therapy; ECOG PS, Eastern Cooperative Oncology Group performance status; EOBD, extent of bone disease; EOD, extent of disease; EORTC, European Organisation for Research and Treatment of Cancer; ePRO-CTCAE, electronic PRO-Common Terminology Criteria for Adverse Events questionnaire; FACT-P, Functional Assessment of Cancer Therapy – Prostate; FAERS, The United States Food and Drug Administration Adverse Events Reporting System; Hb, hemoglobin; HR, hazard ratio; HRD, homologous recombination deficiency; IQR, interquartile range; LDH, lactate dehydrogenase; ^177^Lu-PSMA, lutetium-177-prostate-specific membrane antigen ligand; m, months; mCRPC, metastatic CRPC; MTV, metabolic tumor volume; NHT, novel hormonal therapy; NRS, numerical rating scale; OR, odds ratio; OS, overall survival; PERCIST, PET Response Criteria in Solid Tumors; PET, positron emission tomography; Plt, number of platelets; PROs, patient-reported outcomes; PRR, proportional reporting ratio; PSA, prostate-specific antigen; pts, patients; QoL, quality of life; ^223^Ra, radium-223; ROR, reporting odds ratio; RP, radical prostatectomy; SAEs, serious adverse events; SREs, skeletal-related events; SSEs, symptomatic skeletal events; tALP, total ALP; TAP, time to ALP progression; TEAEs, treatment-emergent adverse events; TRAEs, treatment-related adverse events; TST, time to initiation of subsequent systemic therapy; TTFD, total FACT-P deterioration; TTP, time to pain progression; VAS, visual analog scale; VS, vertebral fractures.

**Supplementary Table 2**. Real-world evidence of safety and tolerability of ^223^Ra

| - **Reference** | - **Country** | - **Number of pts** | - **Study design** | - **Safety outcomes** |
| --- | --- | --- | --- | --- |
| Stattin P, et al. Real World Outcomes in Patients With Metastatic, Castration-Resistant Prostate Cancer Treated With Radium-223 in Routine Clinical Practice in Sweden. *Clin Genitourin Cancer*. 2022;S1558-7673(22)00194-X. doi: 10.1016/j.clgc.2022.09.002 | - Sweden | - 1,434 | Database study | - For ^223^Ra vs. other standard treatments, the difference in 36-month fracture risk was 6% (95% CI, −7–18%) in the first-line cohort (n=635) and 8% (95% CI, −7%–18%) in the second-line cohort (n=453) - Fracture numbers in the third-/fourth-line cohorts were too small to conduct adjusted comparisons |
| Hyväkkä A, et al. Radium-223 dichloride treatment in metastatic castration-resistant prostate cancer in Finland: A real-world evidence multicenter study. *Cancer Med*. 2022;00:1–13. doi: 10.1002/cam4.5262 | - Finland | - 160 | Retrospective, multicenter | - Most patients had Grade 1–2 AEs, but only 12.5% of patients had grade 3–4 AEs - The most common Grade 3–4 AEs were neutropenia (6%), anemia (4%), leukopenia (3%), thrombocytopenia (2%), fatigue and bone pain (both <1%) - One patient experienced fatal thrombocytopenia after ^223^Ra therapy - Twenty-one percent of patients (n=34) discontinued 223Ra due to toxicities |
| Hosono M, et al. Exploratory analysis results from post-marketing surveillance study of radium-223 in Japanese patients with castration-resistant prostate cancer and bone metastases: subgroup analysis by age. *Kaku Igaku.* 2021;58(1):91-101 [In Japanese] | - Japan | - 296 | Post-marketing surveillance study | - The < 75-year-old arm had 148 pts, the ≥75-year-old arm 148 pts, and the ≥ 80-year-old arm 69 pts - The < 75-year-old arm had more aggressive disease at diagnosis and more pts had prior chemotherapy compared with the ≥ 75-year-old arm - The incidences of overall drug-related TEAEs and drug-related hematological TEAEs were slightly higher in the <75-year-old arm |
| Uemura H, et al. Real-world safety and effectiveness of radium-223 in Japanese patients with castration-resistant prostate cancer (CRPC) and bone metastasis: exploratory analysis, based on the results of post-marketing surveillance, according to prior chemotherapy status and in patients without concomitant use of second-generation androgen-receptor axis-targeted agents. *Int J Clin Oncol*. 2021;26(4):753-763. | - Japan | - 296 | Exploratory analyses based on the results of post-marketing surveillance | - The prior-chemotherapy (chemo) arm (n = 126) had higher number of bone metastases, more analgesic use, and higher PSA values than the no prior-chemo arm (n = 170) - Six-month incidence rate of TEAEs, drug-related TEAEs, and grade ≥3 drug-related hematological TEAEs were 47% vs 53%, 25% vs 29%, and 4% vs 7% in the no prior-chemo vs prior-chemo arms - Two lines of prior chemo, Hb, platelet, and LDH were baseline factors significantly related to grade ≥ 2 platelet count decreased - Safety and effectiveness in patients without concomitant ARATs (n = 201) were similar to those in the overall population |
| - Zhao H, et al. Safety of concomitant therapy with radium-223 and abiraterone or enzalutamide in a real-world population.11. Prostate. 2021;81(7):390-397 | - USA | - 318 | - Retrospective chart review,   single center | - 36% of pts received ^223^Ra with concomitant abiraterone/enzalutamide - There was no OS benefit for those on concomitant therapy (HR 0.87, 95% CI 0.67-1.12, P = 0.28) - There was a trend for an increased SRE risk for pts on concomitant therapy (HR 1.87, 95% CI 0.96-3.61, P = 0.066) - When analyses were limited to men using BHA, similar results were seen for OS (HR 0.86, 95% CI 0.64-1.15, p = 0.30) and SRE (HR 2.36, 95% CI 0.94-5.94, P = 0.068) |
| Shore N, et al. Concurrent or layered treatment with radium-223 and enzalutamide or abiraterone/prednisone: real-world clinical outcomes in patients with metastatic castration-resistant prostate cancer. *Prostate Cancer Prostatic Dis.* 2020;23(4):680-688 | - USA, Canada, Belgium, Germany | - 625 | Retrospective using electronic health record data from the Flatiron Health database | - 22% of pts received ^223^Ra together with abiraterone/prednisone and 27% with enzalutamide - 73% of pts layered, 23% concurrent - 67% of pts had prior BHAs and 55% of pts concomitant BHAs - Incidence rates for SSEs and pathologic fractures were 0.35 and 0.11 patients per person-year, respectively |
| Huynh-Le MP, et al. Adverse events associated with radium-223 in metastatic prostate cancer: Disproportionality analysis of FDA data reflecting worldwide utilization. *Clin Genitourin Cancer.* 2020;18(3):192-200 | - Global | - 2182 | Analysis of FAERS | - In pts with ^223^Ra-associated AEs, the median therapy duration was 56 days (around 2-3 treatment cycles) - Disproportionate signals were detected for general health deterioration (ROR 5.03, 95% CI 4.23-5.98; PRR 4.94, 95% CI 4.16-5.87), bone pain (ROR 4.53, 95% CI 3.67-5.59; PRR 4.48, 95% CI 3.63-5.53) and hematologic AEs, including anemia (ROR 2.89, 95% CI 2.55-3.27; PRR 2.80, 95% CI 2.48-3.17), thrombocytopenia (ROR 3.22, 95% CI, 2.77-3.74; PRR 3.16, 95% CI 2.72-3.67) and pancytopenia/bone marrow failure (ROR 4.83, 95% CI 4.11-5.67; PRR 4.73, 95% CI 4.03-5.55) - Pts with mCRPC experiencing AEs receive a half of the prescription dose of ^223^Ra required for survival benefit |
| Raimondi A, et al. Safety and activity of radium-223 in metastatic castration-resistant prostate cancer: the experience of Istituto Nazionale dei Tumori. *Tumori*. 2020;106(5):406-412 | - Italy | - 41 | Retrospective, observational | - Pts received a median of 5 cycles (IQR 3-6) - Any-grade AEs occurred in 73% and grade 3/4 TRAEs in 29% of pts, mainly anemia, decreased platelet count, and fatigue - No SREs or treatment-related deaths |
| Caffo O, et al. Fracture risk and survival outcomes in metastatic castration-resistant prostate cancer patients sequentially treated with abiraterone acetate and RADIUM-223. Eur J Nucl Med Mol Imaging. 2020;47(11):2633-2638 | - Italy | - 94 | Retrospective | - 85.1% of pts received ^223^Ra as second- or third-line treatment - 4 cases of grade 3 anemia, two cases of grade 3 leukopenia and one case of grade 3 neutropenia were reported - The overall fracture rate was 2.1%; both fractures occurred at metastatic sites |
| Prelai A, et al. Radium-223 in patients with metastatic castration-resistant prostate cancer: Efficacy and safety in clinical practice. *Oncol Lett*. 2019;17(2):1467-1476 | - Italy | - 32 | Retrospective, multicenter | - Grade 3-4 toxicities developed in 16% of pts |
| Soldatos TG, et al. Retrospective toxicological profiling of radium-223 dichloride for the treatment of bone metastases in prostate cancer using adverse event data. *Medicina (Kaunas).* 2019 May 16;55(5):149 | - USA | - ~1500 | Retrospective analysis of FAERS | - Cohort A included pts treated with ^223^Ra, and Cohort B pts treated with ^223^Ra and other drugs - Cohorts A: blood count abnormal (PRR=43.82), bone marrow failure (PRR = 12.42), hemoglobin decreased (PRR = 11.07), bone pain (PRR = 10.42), platelet count decreased (PRR = 7.52), neutrophil count decreased (PRR=6.49), pancytopenia (PRR = 5.99), and thrombocytopenia (PRR = 4.80) - Cohort B: bone pain (PRR=17.94), pancytopenia (PRR = 15.24), blood count abnormal (PRR = 14.94), anemia (PRR = 10.44), thrombocytopenia (PRR = 9.36), osteonecrosis (PRR = 9.32), platelet count decreased (PRR = 8.57), decreased appetite (PRR = 6.56), bone marrow failure (PRR = 6.49), and hemoglobin decreased (PRR = 5.71) |
| Skelton WP, et al. A single-center retrospective analysis of the effect of radium-223 (Xofigo) on pancytopenia in patients with metastatic castration-resistant prostate cancer. *Cureus.* 2020;12(1):e6806 | - USA | - 23 | Retrospective, single center | - 73% of pts completed 6 cycles of ^223^Ra - 45% developed pancytopenia - Older age and higher ECOG PS score correlated with increased risk of pancytopenia - A higher proportion of pts who received prior radiation therapy were more likely to develop pancytopenia (90% vs 75%) |
| Kapoor A, et al. Single-center experience with radium-223 in patients with castration-resistant prostate cancer and bone metastases. *Asian J Androl.* 2020;22(4):437-438. | - USA | - 60 | Retrospective | - AE rate was 72%, and grade 3–4 AE rate 42% - The most common AEs were fatigue (41.7%), anemia (21.7%), nausea (28.3%), diarrhea (16.7%), and thrombocytopenia (15.0%) |
| Costa RP, et al. Hematologic toxicity of radium-223 in elderly patients with metastatic castration resistant prostate cancer: a real-life experience. *Prostate Int.* 2019;7(1):25-29 | - Italy | - 38 | Retrospective | - 72.4% of pts had hematological AEs; 36.8% had anemia - Hematological AEs were more common in elderly patients (aged > 75 years) with greater disease burden and previously treated with docetaxel |
| Dizdarevic S, et al. Interim analysis of the REASSURE (Radium-223 alpha Emitter Agent in non-intervention Safety Study in mCRPC popUlation for long-teRm Evaluation) study: patient characteristics and safety according to prior use of chemotherapy in routine clinical practice. *Eur J Nucl Med Mol Imaging.* 2019;46(5):1102-1110 | - Global | - 583 | Global, prospective | - Drug-related TEAEs occurred in 48% of pts - Hematological drug-related TEAEs occurred in 9% of pts who had or had not previously received chemotherapy - Four drug-related deaths were reported, all in the prior chemotherapy group |
| Kuronva Z, et al. A retrospective analysis of the first 41 mCRPC patients with bone pain treated with Radium-223 at the National Institute of Oncology in Hungary. *Pathol Oncol Res.* 2017;23(4):777-783. | - Hungary | - 41 | Retrospective | - The most common side effects were anemia (32% grade 1-3), nausea (28%, grade 1), diarrhea (4%, grade 2), thrombocytopenia (4%, grade 3) |
| Alva A, et al. Clinical correlates of benefit from radium-223 therapy in metastatic castration resistant prostate cancer. *Prostate.* 2017;77(5):479-488 | - USA, Sweden | - 145 | Retrospective | - Grade ≥ 3 neutropenia, anemia, and thrombocytopenia occurred in 4%, 4% and 5% of pts, respectively |
| Song Y, et al. Comparing clinical outcomes for Radium-223: Do older patients do worse? *Int J Radiat Oncol Biol Phys.* 2017;98(4):955-957 | - UK | - 129 | Retrospective, two centers | - There was a higher rate of grade 3 anemia in younger pts |
| Dan TD, et al. Hematologic toxicity of concurrent administration of radium-223 and next-generation antiandrogen therapies. *Am J Clin Oncol.* 2017;40(4):342-347 | - USA | - 25 | Retrospective | - ^223^Ra vs concurrent therapy:   - Mean change from initial neutrophil count to nadir was 1.9×10^6^/L vs 2.3×10^6^/L (P = 0.77)   - Mean change from initial Hb value to nadir was 1.5 g/L vs 1.8 g/L (P = 0.31)   - Mean change from initial platelet count to nadir was 52.3×10^9^ cells/L vs 70.6×10^9^ cells/L (P = 0.39) |
| Trieu J, et al. Lower fracture rates in patients treated with radium-223, abiraterone or enzalutamide, when given concurrently with bone health agents: a real-world analysis. *Clin Genitourin Cancer*. 2022;S1558-7673(22)00092-1. doi: 10.1016/j.clgc.2022.04.015 | - USA | - 177 | Retrospective | - Overall, 159 patients (89%) were on a BHA before and/or during ^223^Ra therapy - 67 patients (38%) received denosumab, 63 (36%) received zoledronic acid, and 29 (16%) received both non-concurrently - Fractures occurred in 11 patients (6.2%) after initiation of ^223^Ra, 9 of which were while the patient was on prior and/or concurrent BHA - The fracture rate in mCRPC patients receiving combination therapy and denosumab or zoledronic acid was 5.7% |
| Van der Doelen MJ, et al. Health-related quality of life, psychological distress, and fatigue in metastatic castration-resistant prostate cancer patients treated with radium-223 therapy. *Prostate Cancer Prostatic Dis*. 2022 Jul 8. doi: 10.1038/s41391-022-00569-8 | - The Netherlands | - 122 |  | - Worse HRQoL (including measures related to bone pain), bone pain intensity, psychological distress and fatigue at baseline and more frequent deterioration of HRQoL, psychological distress and fatigue over time were associated with failure to complete 6 cycles of ^223^Ra treatment - Trajectory analyses have also shown that HRQoL outcomes are likely to worsen over time in patients treated with ^223^Ra with baseline opioid use, and low Hb and high ALP levels |

ACs, academic centers; ADT, androgen deprivation therapy; AEs, adverse events; AIR, annual incidence rate; ALP, alkaline phosphatase; ARATs, androgen-receptor axis-targeted agents; ART, androgen-receptor targeting therapy; BAP, bone-type alkaline phosphatase; BHA, bone health agent; BMI, body mass index; BSI, bone scan index; BST, bone supportive therapy; CAPRI, Castration-resistant Prostate Cancer Registry; CI, confidence interval; CPs, community practices; CRPC, castration-resistant prostate cancer; CT, computed tomography; 1-CTP, serum marker for bone metastasis; DDR, DNA damage repair; EBRT, external beam radiation therapy; ECOG PS, Eastern Cooperative Oncology Group performance status; EOBD, extent of bone disease; EOD, extent of disease; EORTC, European Organisation for Research and Treatment of Cancer; ePRO-CTCAE, electronic PRO-Common Terminology Criteria for Adverse Events questionnaire; FACT-P, Functional Assessment of Cancer Therapy – Prostate; FAERS, The United States Food and Drug Administration Adverse Events Reporting System; Hb, hemoglobin; HR, hazard ratio; HRD, homologous recombination deficiency; IQR, interquartile range; LDH, lactate dehydrogenase; ^177^Lu-PSMA, lutetium-177-prostate-specific membrane antigen ligand; m, months; mCRPC, metastatic CRPC; MTV, metabolic tumor volume; NHT, novel hormonal therapy; NRS, numerical rating scale; OR, odds ratio; OS, overall survival; PERCIST, PET Response Criteria in Solid Tumors; PET, positron emission tomography; Plt, number of platelets; PROs, patient-reported outcomes; PRR, proportional reporting ratio; PSA, prostate-specific antigen; pts, patients; QoL, quality of life; ^223^Ra, radium-223; ROR, reporting odds ratio; RP, radical prostatectomy; SAEs, serious adverse events; SREs, skeletal-related events; SSEs, symptomatic skeletal events; tALP, total ALP; TAP, time to ALP progression; TEAEs, treatment-emergent adverse events; TRAEs, treatment-related adverse events; TST, time to initiation of subsequent systemic therapy; TTFD, total FACT-P deterioration; TTP, time to pain progression; VAS, visual analog scale; VS, vertebral fractures.

**Supplementary Table 3.** Variables associated with survival outcomes in patients with mCRPC treated with ^223^Ra

| - **Reference** | - **Country** | - **Number of pts** | - **Study** | - **Outcomes** |
| --- | --- | --- | --- | --- |
| - Stattin P, et al. Real-World Outcomes in Patients With Metastatic, Castration-Resistant Prostate Cancer Treated With Radium-223 in Routine Clinical Practice in Sweden. *Clin Genitourin Cancer*. 2022;S1558-7673(22)00194-X. doi: 10.1016/j.clgc.2022.09.002 | - Sweden | - 1,434 | - Database study | - 36-month mortality was higher in the first-line cohort 13% (95% CI, −3–31%), but lower in the second- and third-/fourth-line cohorts −8% (95% CI, −23–7%) and −14% (95% CI, −21–16%), respectively - Most deaths were due to prostate cancer |
| - Hyväkkä A, et al. Radium-223 dichloride treatment in metastatic castration-resistant prostate cancer in Finland: A real-world evidence multicenter study. *Cancer Med*. 2022; doi: 10.1002/cam4.5262 | - Finland | - 160 | - Retrospective, multicenter | - Median OS 13.8 months (range 0.5–57 months) - Pain relief was prognostic for OS (overall P=0.002; median OS, 16.0 [pain/use of analgesics decreased] vs. 14.0 [pain/use of analgesics stayed the same] vs. 9.4 [pain/use of analgesics increased] months) - OS improvement in patients achieving ALP normalization to reference values vs. patients with elevated ALP values before and during treatment (median OS 17.3 vs. 9.1 months, p < 0.0001) - Longer OS associated with lower baseline PSA (overall P<0.0001) - PSA decrease during ^223^Ra treatment associated with longer OS vs. PSA increase (median OS 23.3 vs. 13.4 months, P=0.0003) - More ^223^Ra cycles completed associated with longer OS (overall P<0.0001) - Shorter time between metastases to ^223^Ra initiation associated with shorter OS (overall P<0.01) - Better ECOG PS associated with better OS (overall P=0.01) - No significant association between OS and line of ^223^Ra therapy |
| - Bauckneht M, et al. The prognostic power of inflammatory indices and clinical factors in metastatic castration-resistant prostate cancer patients treated with radium-223 (BIO-Ra study). *Eur J Nucl Med Mol Imaging*. 2022;49(3):1063-1074 | - Italy | - 519 | - Real-world, multicenter | - Median OS was 19.9 m - Higher NLR, dNLR, PLR, and SII and lower LMR predicted worse OS (all with a P < 0.001) - The multivariable model including NLR, ECOG PS, number of bone metastases, ALP, and PSA (c-index: 0.724) was chosen to develop the BIO-Ra score - The BIO-Ra score identified three prognostic groups with median OS of 31, 26.6, and 9.6 m, respectively (HR 1.62, P=0.008 for group 2 vs 1 and 5.77, P < 0.001 for group 3 vs 1) |
| - Sasaki D, et al. Effects of six-cycle completion and earlier use of radium-223 therapy on prognosis for metastatic castration-resistant prostate cancer: A real-world multicenter retrospective study. *Urol Oncol.* 2022;40(2):64.e1-64.e8 | - Japan | - 75 | - Retrospective | - Median number of ^223^Ra cycles was 6 (IQR 5–6) - Median ^223^Ra completion 75% - Unfavorable ECOG PS (> 0), PSA > 10 ng/ml, extension of bone metastasis score 3 to 4, and incomplete ^223^Ra cycles were significantly associated with poor OS - EOD 3–4 and ≥ 3 prior CRPC treatments were significantly associated with incomplete number of ^223^Ra cycles |
| Zhao H, et al. Racial discrepancies in overall survival among men treated with (223) radium. *J Urol.* 2020;203(2):331-337 | - USA | - 318 | Chart review of the Veterans Affairs system | - 27% of pts were black - Median follow-up after ^223^Ra initiation was 25.3 m (IQR 13.8–37.1) - Black pts had higher baseline PSA (median 159.9 vs 90.2 ng/ml, P = 0.014) and ALP (median 163 vs 135 IU/l, P = 0.017) than nonblack men - 77% of black men received prior docetaxel vs 55% of nonblack pts (P < 0.001) - Black race was associated with a decreased risk of mortality from the start of ^223^Ra (HR 0.75, 95% CI 0.57-0.99, P = 0.045) |
| Maruzzo M, et al. Results from a large, multicenter, retrospective analysis on radium223 use in metastatic castration-resistant prostate cancer (mCRPC) in the Triveneto italian region. *Clin Genitourin Cancer.* 2019;17(1):e187-e194 | - Italy | - 158 | - Retrospective, multicenter | - Median follow-up was 9.5 m - Median OS was 14.2 m, and PFS 6.2 m - 45% of pts achieved progression as best response - ECOG PS (0–2) was prognostic for OS (18.4 vs 12.3 vs 7.5 m; 0 vs 1, P = 0.0062; 0 vs 2, P = 0.0002) - A NLR ≥3 significantly impacted OS (18.1 vs 9.7 m; P < 0.001) and slightly impacted PFS (6.6 vs 5.6 months; P = 0.05) - Pts with a baseline ALP value ≥ 220 U/L had worse OS and PFS (24.1 vs 10.5 m; 7.2 vs 5.5 m; P < 0.001) - Pts with changes in ALP value achieved better OS (P = 0.029) and PFS (P = 0.002) - The main grade 3-4 toxicities were anemia, asthenia, and thrombocytopenia |
| Parikh S, et al. Real-world outcomes and factors predicting survival and completion of radium 223 in metastatic castrate-resistant prostate cancer. *Clin Oncol (R Coll Radiol).* 2018;30(9):548-555 | - UK | - 189 | - Retrospective cohort study, multicenter | - Median OS was 10.5 m - Pts treated with 6 ^223^Ra cycles had OS of 18.6 m - Independent predictors of OS were age (P = 0.005, HR 1.07, 95% CI 1.02–1.12); 5–6 vs 1–4 ^223^Ra cycles (P ≤ 0.001, HR 0.10, 95% CI 0.005–0.20); baseline ALP (P = 0.044, HR 1.06, 95% CI 1.002–1.12); NLR (P = 0.033, HR 1.19, 95% CI 1.01–1.40) - Baseline ECOG PS 0 vs 2 (P = 0.026), OR 0.080 (95% CI 0.001–0.74) and higher baseline Hb (P = 0.028), OR 1.04 (95% CI 1.004–1.074) were independent predictors of the completion of 5–6 ^223^Ra cycles |
| George DJ, et al. Real-world patient characteristics associated with survival of 2 years or more after radium-223 treatment for metastatic castration-resistant prostate cancer (EPIX study). *Prostate Cancer Prostatic Dis*. 2022 doi: 10.1038/s41391-021-00488-0 | - US | - 1180 | - Retrospective chart review of Flatiron database | - Median OS was 12.9 months (95% CI 12.1–13.7) - At 6 months, 13% of pts had a PSA response - 65.7% of pts survived < 2 years, 15.7% survived ≥ 2 years - Age > 75 years, ECOG PS 2–4, visceral metastases, prior symptomatic SSEs, and prior chemotherapy were independently prognostic of reduced OS |
| Augusti T, et al. Prognostic factors in metastatic castration resistant prostate cancer patients treated with Radium-223: a retrospective study. *Minerva Urol Nephrol.* 2022 doi: 10.23736/S2724-6051.22.04701-2. Online ahead of print | - Italy | - 75 | - Retrospective, single center | - Lymph node involvement (HR 1.68, 95% CI 1.01–2.80, P = 0.047), absence of local treatment on primary tumor (HR 1.93, 95% CI 1.13–3.29, P = 0.016), baseline strong opioids use (HR 1.82, 95% CI 1.08–3.06, P = 0.024), high PLR (HR 1.91, 95% CI 1.06–3.45, P = 0.03), high baseline ALP (HR 1.81, 95% CI 1.10–2.99, P = 0.019) and high baseline LDH (HR 3.86, 95% CI 2.01–7.41, P < 0.001) were significantly associated with worst OS |
| Charrois-Durand C, et al. A single center, multidisciplinary experience with radium-223 dichloride in men with metastatic castrate-resistant prostate cancer. *Can Urol Assoc J.* 2022. doi: 10.5489/cuaj.7591. Online ahead of print | - Canada | - 133 | - Real-world, single center | - Median OS was 9.0 m - Using baseline Hb, tALP, and ECOG PS, patients were divided into three groups with different median OS (m): 23.0 (95% CI 12.8–33.2), 8.0 (95% CI 6.7–9.3) and 5.0 (95% CI 3.1–6.9) |
| AL-Ezzi EM, et al. Clinicopathologic factors that influence prognosis and survival outcomes in men with metastatic castration-resistant prostate cancer treated with Radium-223. *Cancer Med.* 2021;10(17):5775-5782 | - Canada | - 150 | - Retrospective, single center | - 63% of pts received > 4 ^223^Ra doses, 37% received ≤ 4 - The median OS for all pts was 14.5 m (95% CI 11.2–18) - Factors that were associated with poor survival outcomes in UVA and MVA were ALB < 35 g/L, ALP > 150 U/L, ECOG PS 2–3, and PSA > 80 µg/L |
| Van der Doelen M, et al. Early alkaline phosphatase dynamics as biomarker of survival in metastatic castration-resistant prostate cancer patients treated with radium-223. *Eur J Nucl Med Mol Imaging*. 2021;48(10):3325-3334 | - The Netherlands | - 180 | - Retrospective, multicenter | - Median OS was 13.5 m (95% CI 11.5–15.5) - Pts with elevated baseline ALP without ALP response after the first injection had significantly worse OS when compared with all other pts (median OS 7.9 m vs 15.7 m; P < 0.001) - Pts with elevated baseline ALP without ALP response after the first injection had significantly shorter times to ALP progression and first SRE, and more frequently discontinued ^223^Ra therapy when compared with other pts |
| Frantellizzi V, et al. Validation of the 3-variable prognostic score (3-PS) in mCRPC patients treated with (223) Radium-dichloride: a national multicenter study. *Ann Nucl Med.* 2020;34(10):772-780 | - Italy | - 430 | - Retrospective, multicenter | - The 3-variable prognostic score includes baseline Hb, PSA and ECOG PS was valid on the total group (N=430) and in the group (N = 338) without 92 pts who were enroled in the original study - The score can select pts who will most probably complete ^223^Ra treatment |
| van der Doelen MJ, et al. Impact of DNA damage repair defects on response to radium-223 and overall survival in metastatic castration-resistant prostate cancer. *Eur J Cancer.* 2020; 136:16-24 |  | - 93 | - Retrospective | - 30% of pts had DDR mutations - DDR+ pts showed prolonged OS (median 36.3 vs 17.0 m; HR 2.29; P = 0.01) - Median TAP and TST in the DDR+ and DDR- patients was 6.9 vs 5.8 months (HR = 1.48; P = 0.15), and 8.9 vs 7.3 months (HR = 1.58; P = 0.08), respectively - DDR+ patients more frequently completed ^223^Ra therapy (79% vs 47%; P = 0.05) - No difference in biochemical response |
| Mazziotti G, et al. Morphometric vertebral fractures in patients with castration-resistant prostate cancer undergoing treatment with radium-223: a longitudinal study in the real-life clinical practice. *Endocrine.* 2020;69(1):204-211 | - Italy | - 49 | - Retospective | - VS significantly associated with duration of ADT (OR 1.29) and previous abiraterone therapy (OR 3.80) in 49% of pts - During ^223^Ra therapy, incident VFs occurred in 25% of pts, in relationship with prevalent VFs (HR 6.89) and change in serum tALP values (HR 0.97), but correlations with ADT and abiraterone therapy were lost - The risk of VFs did not correlate with the therapeutic end points of ^223^Ra |
| Frantellizzi V, et al. Baseline quality of life predicts overall survival in patients with mCRPC treated with (223) Ra-dichloride. *Hell J Nucl Med.* 2020;23(1):12-20. | - Italy | - 173 | - Prospective | - Baseline Hb, tALP and QoL-score were independent significant predictors of OS (QoL score: HR 0.99, 95% CI 0.992–0.998, P = 0.001) - The OS analysis stratified by score of baseline QoL, showed a median OS of 8 m (95% CI 6–11) and 16 m (95% CI 12–24) for scores respectively below and above the cut-off value (log-rank-P < 0.001) |
| Vidal M, et al. Overall survival prediction in metastatic castration-resistant prostate cancer treated with radium-223. *Int Braz J Urol.* 2020;46(4):599-611. | - Colombia | - 40 | - Prospective | - Median OS was 17.1 m - 26/40 pts received complete treatment with ^223^Ra and did not reach a median OS - 14/40 pts received incomplete treatment with a median OS 13.6 m - VAS, ECOG, Hb and ALP were independently associated with OS |
| Frantellizzi V, et al. Primary radical prostatectomy or ablative radiotherapy as protective factors for patients with mCRPC treated with radium-223 dichloride: An Italian multicenter study. *Clin Genitourin Cancer.* 2020;18(3):185-191. | - Italy | - 275 | - Retrospective, multicenter | - 48% were in the RP/EBRT group - Median survival in RP/EBRT group was 18 m vs 11 m in no RP/EBRT group (P < 0.001) |
| Gazzilli M, et al. Three years' clinical practice of Radium-223 therapy in patients with symptomatic bone metastases from metastatic castrate-resistant prostate cancer: a single-centre experience. *Nucl Med Commun.* 2020;41(4):300-307 | - Italy | - 38 | - Retrospective | - Pain reduction occurred in 16 pts, did not change in 14 pts and increased in 8 pts - Pts with < 10 vs > 10 metastasis had significant differences in PFS (P < 0.001) - OS was similar between the groups (P = 0.23) |
| Badrising SK, et al. A prospective observational registry evaluating clinical outcomes of radium-223 treatment in a non-study population. *Int J Cancer.* 2020;147(4):1143-1151 | - The Netherlands | - 300 | - Prospective registry, multicenter | - 90% of pts had ≥6 bone metastases, 74.1% were pre-treated with docetaxel, 19.5% with cabazitaxel and 80.5% with abiraterone and/or enzalutamide - 96.7% were treated with ^223^Ra (median of 5 cycles) - 6-month SSE-free survival rate was 83%, median PFS 5.1 m and median OS 15.2 m - "Previous cabazitaxel treatment" and "bone-only metastases" were independent predictors of a shorter and longer PFS, respectively - Above-median LDH and "bone-only metastases" were independent predictors of shorter and longer OS, respectively |
| Bauckneht M, et al. Role of baseline and post-therapy 18F-FDG PET in the prognostic stratification of metastatic castration-resistant prostate cancer (mCRPC) patients treated with radium-223. Cancers (Basel). 2019;12(1):31 | - Italy | - 28 | - Retrospective | - At baseline, PSA, LDH, and MTV significantly predicted OS - MTV (but not PSA nor LDH) identified a subgroup of pts with worse prognosis - After therapy, PERCIST criteria demonstrated longer OS in pts with partial metabolic response |
| Nakashima K, et al. Initial experience with radium-223 chloride treatment at the Kanazawa University Hospital. *Anticancer Res.* 2019;39(5):2607-2614 | - Japan | - 26 | - Retrospective | - Fatigue and nausea were main AEs - ALP and BAP levels decreased following the treatment; PSA and 1-CTP increased - OS was significantly improved when baseline BSI value was < 2 compared with ≥ 2 - Decrease in BSI after ^223^Ra was an independent factor for significantly prolonged OS |
| Roviello G, et al. Pain predicts overall survival in men with metastatic castration-resistant prostate cancer treated with radium-223. *Onco Targets Ther.* 2018; 12:9-13 | - Italy | - 25 | - Single center | - Median OS was 8.3 m (95% CI 5.2–11.8) - Pain was assessed using a VAS - Median OS was 12.6 m in pts with VAS < 4 vs 6.6 m in pts with VAS ≥ 4 (P = 0.03) |
| Isaacsson Velho P, et al. Efficacy of Radium-223 in Bone-metastatic castration-resistant prostate cancer with and without homologous repair gene defects. *Eur Urol*. 2019;76(2):170-176 | - US | - 28 | - Retrospetive, single center | - 10 pts had HRD mutations, 18 pts did not - Comparing HRD+ with HRD- pts, HRD+ showed greater ALP responses (80% vs 39%, P = 0.04), longer time to ALP progression (10.4 vs 5.8 m, P = 0.005), and longer OS (median 36.9 vs 19.0 m, P = 0.11) |
| Rathburn J and Franklin G. Radium-223 (Xofigo) with concurrent abiraterone or enzalutamide: predictive biomarkers of improved overall survival in a clinically advanced cohort. *Curr Probl Cancer.* 2019;43(3):205-212 | - US | - 35 | - Retrospective | - Median cohort OS was 10 m - Pts with no or mild pain had longer median OS than those with moderate or severe pain, 14 m vs 7 m (P = 0.028) - Pts with ECOG PS < 2 had longer median OS than those with ECOG PS ≥ 2, 13 m vs 10 m (P = 0.0233) |
| Leisser A, et al. Analysis of hematological parameters as prognostic markers for toxicity and survival of (223) radium treatment. *Oncotarget.*2018;9(22):16197-16204 | - Austria | - 54 | - Retrospective | - Median OS was 67 weeks - In pts with pre-existing grade 2 Hb toxicity, median OS was 48 weeks vs 67 weeks for grade 1 and not reached for normal Hb levels |
| Parimi S, et al. A population-based study of the use of radium 223 in metastatic castration-resistant prostate cancer: Factors associated with treatment completion. *Can Urol Assoc J.* 2017;11(10):350-355 | - Canada | - 91 | - Retrospective | - Median OS was 10.7 m - PSA and ALP >30% decline from baseline occurred in 21% and 52% of pts, respectively - Pts with ≥ 5 ^223^Ra cycles had longer median OS than those with < 5 cycles (16.2 vs 5.9 m; P < 0.0001) |
| Leisser A, et al. Hematopoiesis is prognostic for toxicity and survival of (223) Radium treatment in patients with metastatic castration-resistant prostate cancer. *Hell J Nucl Med*. 2017;20 Suppl:157 | - Austria | - 56 | - Retrospective | - Median OS was 69.9 weeks - Pts with thrombocytopenia had a significantly shorter survival compared with those with normal Plt levels (21 weeks vs not reached; P < 0.003) - Pts with pre-therapeutic low Hb-level (< 10g/dL) had a significantly shorter survival compared with those with Hb > 10 g/dL (28 weeks vs not reached, P < 0.004) - Pts with impaired Hb also developed significantly more grade 3 and 4 HT (Hb < 10 g/dL: 42.9 vs 14.3%, P < 0.001; Plt < 150 g/mL: 25.0% vs 6.3%; P = 0.002) - Pts with impaired Hb received significantly fewer treatment cycles (Hb < 10g/dL: 5.1 vs 5.8, P < 0.04; Plt < 150g/mL: 3.4 vs 5.6; P < 0.001) |
| Frantellizzi V, et al. A 3-variable prognostic score (3-PS) for overall survival prediction in metastatic castration-resistant prostate cancer treated with (223) Radium-dichloride. *Ann Nucl Med.* 2018;32(2):142-148 | - Italy | - 92 | - Retrospective, single center | - Patients' weight, BMI, ECOG PS, Hb and tALP values were independently associated with OS - In multivariable analysis, only baseline Hb and ECOG PS remained significantly correlated with OS - Combination of baseline ECOG PS with Hb < 12 g/dl and PSA ≥ 20 ng/ml resulted in a score 0–4 (P < 0.001) |
| De Luca R, et al. The clinical efficacy of radium-223 for bone metastasis in patients with castration-resistant prostate cancer: An Italian clinical experience. *Oncology.* 2018;94(3):161-166 | - Italy | - 48 | - Retrospective | - ^223^Ra was well tolerated, with a manageable toxicity profile and a modest objective response rate - A considerable difference in serum ALP levels before and after treatment was observed, with a significant correlation between pain relief and QoL (P = 0.0021) |
| Van der Doelen M, et al. 223Ra therapy in patients with advanced castration-resistant prostate cancer with bone metastases: Lessons from daily practice. *Clin Nucl Med.* 2018;43(1):9-16 |  | - 45 | - Retrospective | - 47% of pts received 6 ^223^Ra injections and 53% received 1-5 ^223^Ra injections - Median OS from start of ^223^Ra treatment was 13.0 m - Pts with 6 ^223^Ra cycles had a median OS of 19.7 m - Pts with 1-5 ^223^Ra injections had a median OS of 5.9 m |
| Fosbøl M, et al. Impact of treatment delay in Radium-223 therapy of metastatic castration-resistant prostate cancer patients. *Ann Nucl Med*. 2018;32(1):16-21 | - Denmark | - 50 | - Retrospective, single center | - 34% of pts had prolonged intervals between cycles (median delay 4 weeks) - Pts with delayed treatment had significantly longer median rPFS (7.1 m vs 4.5 m) - There was no significant difference in number of completed cycles or median OS |
| Fosbøl M, et al. 223Ra Therapy of advanced metastatic castration-resistant prostate cancer: Quantitative assessment of skeletal tumor burden for prognostication of clinical outcome and hematologic Toxicity*. J Nucl Med.* 2018;59(4):596-602 | - Denmark | - 88 | - Retrospective | - Median OS for pts with BSI of > 5 was 8.2 m, and for pts with BSI of ≤ 5 was 15.0 m (P = 0.001) - Pts with BSI of > 5 had OR of 3.02 for toxicity |
| Kawahara T, et al. Administration of radium-223 and the prognosis in Japanese bone metastatic castration-resistant prostate cancer patients: A large database study. *Int J Urol*. 2022 Aug 17. doi: 10.1111/iju.15008 | - Japan | - 25,934 | - Retrospective | - Patients completing 6 cycles of ^223^Ra had significantly better OS and cancer-specific survival versus those who received ≤5 cycles (P < 0.0001 and P < 0.0001, respectively) - In patients with bone metastatic CRPC, OS and cancer-specific survival were significantly improved with ^223^Ra versus those who did not receive ^223^Ra (P < 0.0001 and P < 0.0001, respectively) |

Abbreviations

ACs, academic centers; ADT, androgen deprivation therapy; AEs, adverse events; AIR, annual incidence rate; ALP, alkaline phosphatase; ARATs, androgen-receptor axis-targeted agents; ART, androgen-receptor targeting therapy; BAP, bone-type alkaline phosphatase; BHA, bone health agent; BMI, body mass index; BSI, bone scan index; BST, bone supportive therapy; CAPRI, Castration-resistant Prostate Cancer Registry; CI, confidence interval; CPs, community practices; CRPC, castration-resistant prostate cancer; CT, computed tomography; 1-CTP, serum marker for bone metastasis; DDR, DNA damage repair; dNLR, derived-neutrophil to lymphocyte ratio; EBRT, external beam radiation therapy; ECOG PS, Eastern Cooperative Oncology Group performance status; EOBD, extent of bone disease; EOD, extent of disease; EORTC, European Organisation for Research and Treatment of Cancer; ePRO-CTCAE, electronic PRO-Common Terminology Criteria for Adverse Events questionnaire; FACT-P, Functional Assessment of Cancer Therapy – Prostate; FAERS, The United States Food and Drug Administration Adverse Events Reporting System; Hb, hemoglobin; HR, hazard ratio; HRD, homologous recombination deficiency; IQR, interquartile range; LDH, lactate dehydrogenase; LMR, lymphocyte to monocyte; ^177^Lu-PSMA, lutetium-177-prostate-specific membrane antigen ligand; m, months; mCRPC, metastatic CRPC; MTV, metabolic tumor volume; NHT, novel hormonal therapy; NLR, neutrophil to lymphocyte ratio; NRS, numerical rating scale; OR, odds ratio; OS, overall survival; PERCIST, PET Response Criteria in Solid Tumors; PET, positron emission tomography; PLR, platelet to lymphocyte ratio; Plt, number of platelets; PROs, patient-reported outcomes; PRR, proportional reporting ratio; PSA, prostate-specific antigen; pts, patients; QoL, quality of life; ^223^Ra, radium-223; ROR, reporting odds ratio; RP, radical prostatectomy; SAEs, serious adverse events; SII, immune-inflammation index; SREs, skeletal-related events; SSEs, symptomatic skeletal events; tALP, total ALP; TAP, time to ALP progression; TEAEs, treatment-emergent adverse events; TRAEs, treatment-related adverse events; TST, time to initiation of subsequent systemic therapy; TTFD, total FACT-P deterioration; TTP, time to pain progression; VAS, visual analog scale; VS, vertebral fractures.
